# Effects of a Real-Life Park-Based Physical Activity Interventional Program on Cardiovascular Risk and Physical Fitness

**DOI:** 10.5888/pcd18.200115

**Published:** 2021-02-25

**Authors:** Bruno Temoteo Modesto, Teresa Bartholomeu, Luciano Basso, Luiz Augusto Riani Costa, Tais Tinucci, Cláudia Lúcia de Moraes Forjaz

**Affiliations:** 1 Exercise Hemodynamic Laboratory, School of Physical Education and Sport, University of São Paulo, São Paulo, Brazil; 2 Motor Behavior Laboratory, School of Physical Education and Sport, University of São Paulo, São Paulo, Brazil

## Abstract

**Introduction:**

Regular physical activity (PA) practice is a way to combat cardiovascular disease, and a PA interventional program, including individualized prescription of walking with limited supervision of execution, may be a strategy to be applied in public parks. Thus, our study tested the effects of a real-world program like this on cardiovascular risk and cardiorespiratory fitness (CF) of the users of a public park.

**Methods:**

Data came from the Exercise and Heart Project, a real-life park-based PA interventional program. The study phases were 1) a preintervention evaluation; 2) the individualized prescription of PA; 3) the supervision of the first practice sessions; 4) the unsupervised execution of the prescription; and 5) a postintervention evaluation.

**Results:**

Data from 152 participants (mainly women and aged 40 to 80 years) were analyzed. The intervention significantly increased CF (mean [standard deviation], 99 [19] steps vs 110 [21] steps, *P* < .001) and reduced body mass index, waist circumference, and systolic blood pressure, decreasing global cardiovascular risk (mean [standard deviation], 0.15 [2.84] vs −0.52 [2.60]; *P* < .001). The effects of intervention on cardiovascular risk were not different between the participants with low and high initial CF or PA levels.

**Conclusion:**

The proposed real-life park-based PA interventional program decreased cardiovascular risk of the participants independently of their initial PA or CF levels.

**Summary**

**What is already known on this topic?**

Increasing physical activity is a way to reduce cardiovascular risk.

**What is added by this report?**

A real-life park-based interventional program including individualized prescription of walking and limited supervision of execution decreases cardiovascular risk and improves cardiorespiratory fitness in users of a public park.

**What are the implications for public health practice?**

The implementation of real-life park-based interventional programs like the one proposed in this study (individualized prescription of walking with limited supervision of execution) may be a strategy to improve cardiovascular health in users of public parks.

**MEDSCAPE CME**

In support of improving patient care, this activity has been planned and implemented by Medscape, LLC and *Preventing Chronic Disease*. Medscape, LLC is jointly accredited by the Accreditation Council for Continuing Medical Education (ACCME), the Accreditation Council for Pharmacy Education (ACPE), and the American Nurses Credentialing Center (ANCC), to provide continuing education for the healthcare team.

Medscape, LLC designates this Journal-based CME activity for a maximum of 1.00 AMA PRA Category 1 Credit(s)™. Physicians should claim only the credit commensurate with the extent of their participation in the activity.

Successful completion of this CME activity, which includes participation in the evaluation component, enables the participant to earn up to 1.0 MOC points in the American Board of Internal Medicine’s (ABIM) Maintenance of Certification (MOC) program. Participants will earn MOC points equivalent to the amount of CME credits claimed for the activity. It is the CME activity provider’s responsibility to submit participant completion information to ACCME for the purpose of granting ABIM MOC credit.

**Release date: February 25, 2021; Expiration date: February 25, 2022**

**Learning Objectives**

Upon completion of this activity, participants will be able to:

1. Distinguish cardiometabolic markers improved by a simple exercise prescription
2. Analyze how baseline levels of physical activity affected the response to exercise in the current study
3. Assess the effects of the exercise intervention in the current study on cardiorespiratory fitness
4. Analyze how baseline levels of cardiorespiratory fitness affected the response to exercise in the current study

**EDITOR**

Caran Wilbanks

Editor

Preventing Chronic Disease

Disclosure: Caran Wilbanks has disclosed no relevant financial relationships.

**CME AUTHOR**

Charles P. Vega, MD

Health Sciences Clinical Professor of Family Medicine

University of California, Irvine School of Medicine

Disclosure: Charles P. Vega, MD, has disclosed the following relevant financial relationships:

Served as an advisor or consultant for: GlaxoSmithKline

**AUTHORS**

**Bruno Temoteo Modesto, MsC**

Exercise Hemodynamic Laboratory

School of Physical Education and Sport

University of São Paulo

São Paulo, Brazil

Disclosure: Bruno Temoteo Modesto, MsC, has disclosed no relevant financial relationships.

**Teresa Tartholo Bartholomeu**

Exercise Hemodynamic Laboratory

School of Physical Education and Sport

University of São Paulo

São Paulo, Brazil

Disclosure: Teresa Tartholo Bartholomeu has disclosed no relevant financial relationships.

**Luciano Basso, PhD**

Exercise Hemodynamic Laboratory

School of Physical Education and Sport

University of São Paulo

São Paulo, Brazil

Disclosure: Luciano Basso, PhD, has disclosed no relevant financial relationships.

**Luiz Augusto Riani Costa, MD**

Exercise Hemodynamic Laboratory

School of Physical Education and Sport

University of São Paulo

São Paulo, Brazil

Disclosure: Luiz Augusto Riani Costa, MD, has disclosed no relevant financial relationships.

**Tais Tinucci, PhD**

Exercise Hemodynamic Laboratory

School of Physical Education and Sport

University of São Paulo

São Paulo, Brazil

Disclosure: Tais Tinucci, PhD, has disclosed no relevant financial relationships.

**Cláudia Lucia de Moraes Forjaz, PhD**

Exercise Hemodynamic Laboratory

School of Physical Education and Sport

University of São Paulo

São Paulo, Brazil

Disclosure: Cláudia Lucia de Moraes Forjaz, PhD, has disclosed no relevant financial relationships.

## Introduction

Cardiovascular diseases are responsible for 19 million deaths per year worldwide (1). In Brazil, they affect 368,000 persons per year and are responsible for 31% of the deaths (2). Cardiovascular diseases are associated with some risk behaviors, such as physical inactivity (3). Thus, promotion of physical activity (PA) is a priority because a clear inverse relationship exists between cardiovascular mortality and PA (4) or cardiorespiratory fitness (CF) (5).

To spread the benefits of PA, health campaigns encourage PA following general prescriptions (6). Consequently, many individuals use public parks for PA (7). However, inadequate PA may acutely trigger cardiovascular events, especially in participants with high cardiovascular risk (5), who represent the greatest portion of the parks’ users (8). Additionally, prescription of PA based on the FITT principle (frequency, intensity, time, and type) induces greater improvements (9). Thus, it is necessary to develop interventions that reduce acute cardiovascular risk, potentiate health benefits, and allow the attendance of many individuals.

An intervention including individualized prescription of PA and limited supervision of execution may address these necessities. Additionally, walking may be an ideal modality because of its easy execution and low cost and risk (10). Previous studies showed that supervised PA in public places (11) and unsupervised programs conducted under experimental conditions (12) promote health benefits. However, no study has evaluated a program with an individualized prescription and limited supervision.

Thus, our study analyzed the effects of a real-life park-based PA interventional program with individualized prescription of walking and limited supervision of practice on cardiovascular risk and CF. Additionally, as responses to PA may depend on the initial levels of PA and CF (13), which vary considerably among park users (14), this study also explored whether the effects of the proposed intervention differ among participants with low and high initial levels of PA and CF.

## Methods

This is a study with a noncontrolled pre–post design that used data from the Exercise and Heart Project. This is a real-life park-based PA interventional program, initiated in 2001 and conducted in Fernando Costa park in São Paulo, Brazil. The project was designed to improve cardiovascular health of the adult users of the park, and participation was free and open to all users of the park (15). Research protocol and informed consent related to this project were approved by the Ethics Committee of the School of Physical Education and Sport of the University of São Paulo (number 10/2002).

Data used in our study were from participants of the Exercise and Heart Project who fulfilled the following criteria: 1) joined the project between October 2001 and October 2015; 2) were aged 40 to 80 years; 3) had signed the informed consent form; 4) had carried out preevaluations and postevaluations within 3 to 6 months; and 5) had reported to have followed, in whole or in part, the PA prescription. Exclusion criteria were 1) presence of cardiovascular symptoms without investigation; 2) presence of cardiovascular disease without physician’s authorization to exercise without supervision; 3) presence of 2 or 3 uncontrolled cardiovascular risk factors according to the Brazilian Metabolic Syndrome guidelines (16) without a physician visit for more than 6 months; and 4) change in medication between preintervention and postintervention evaluations.

Participation included a preintervention evaluation, the individualized prescription of PA, the supervision of the first practice sessions, the unsupervised execution of the prescription, and a postintervention evaluation. All procedures were based on the recommendations of the American College of Sports Medicine guidelines (9).

### Preintervention evaluation

Preintervention evaluation was composed of an interview about health status and PA practice, measurements of cardiovascular risk factors, and a CF assessment. Evaluations were conducted by qualified trained staff and scheduled in advance. The participants were asked to arrive in the morning after a 12-hour overnight fast and without doing any vigorous PA during the previous day.

The interview assessed 1) personal data — name, sex, age, address, and other factors; 2) known health status — presence of cardiovascular disease, symptoms, or risk factors as well as use of cardiovascular medications; and 3) leisure time PA — they were asked if they perform any PA during leisure time; in the affirmative cases, they were asked about which activities they do, and for each activity they were asked about the weekly frequency and the duration of each session.

The cardiovascular risk measurement included 1) anthropometric measures — body weight and height (portable scale with stadiometer, Welmy, model 110), waist circumference (tape positioned at the navel), and body mass index (BMI) calculated as weight in kilograms divided by the square of height in meters; 2) hemodynamic measurements — blood pressure (3 consecutive measures after 5 min of seated rest, using an appropriate cuff) and heart rate (triplicate measure by radial palpation for 15 seconds); and 3) metabolic measurements — fasting glucose and total cholesterol in finger blood samples (Roche Advantage II and Accutrend GC).

CF was assessed by the 2-minute step-test in which the number of full steps completed in 2 min was measured (17). This test was not performed in participants who had resting blood pressure above 160/105 mm Hg.

### Individualized prescription

Individualized PA prescription consisted of the recommendation of walking at least 3 times a week for at least 30 minutes at an intensity between 50% and 70% of the heart rate reserve for inactive and insufficiently active participants, and between 60% and 80% for active and very active participants (using Karvonen’s formula [9]). For heart rate reserve calculation (maximal minus resting heart rate), maximal heart rate was based on participant’s age (220 minus the participant’s age) or on peak heart rate achieved in a maximal test (when participants had this test). For those who could not measure heart rate during PA execution, intensity was prescribed based on the subjective perception of breath (walk as fast as possible without panting and be able to speak a long phrase without interruption to breath). Additionally, participants were instructed to do stretching exercises before and after walking. For that, they received a folder with 12 stretching exercises and were invited to 20-minute stretching classes offered in the park 3 times per week. These classes were a strategy to call participants and to increase their adherence.

### Supervision of the first practice sessions

To ensure the correct execution of the prescribed walking, the first 2 to 3 training sessions were supervised. In these sessions, the participants executed the walking prescription by using a heart rate monitor (Polar Electro Oy, model A3) and were accompanied side-by-side by a supervisor. They measured their heart rate at regular intervals by pulse palpation and the results were checked by comparison with the heart rate monitor. The supervisor taught how to measure heart rate and to change speed to keep intensity within the desired range. For those who were not able to measure heart rate, the supervisor instructed them on how to control intensity by perception of breath. Finally, the supervisor taught the stretching exercises.

### Execution of the prescription

Participants were instructed to do the walking prescription on their own and to do the stretching exercises either on their own or participating in the stretching classes. Execution of stretching exercises was not registered.

### Postintervention evaluation

After 3 to 6 months, participants were invited for the postintervention evaluation. They were asked about whether they had had any change in their health status, medication, or PA. They were also asked if they had followed all exercise prescription parameters: weekly frequency, duration, and intensity. Those who followed all the parameters or part of them (eg, reported to have followed frequency and duration but not intensity) were included in the analyses. Those who reported not to have followed any parameter were excluded. Afterwards, cardiovascular risk factors and CF were reassessed, following the procedures described for the preintervention evaluation.

### Data analysis

PA level was assessed as the total weekly volume calculated as the sum of walking and all reported PA volumes (weekly frequency multiplied by duration). The participants were classified as 1) inactive, 0 minutes per week; 2) insufficiently active, 1 to 149 minutes per week; 3) active, 150 to 299 minutes per week; and 4) very active, 300 or more minutes per week (6). CF level assessed by the 2-minute step-test was classified based on quartiles of the total sample as follows: quartile 1, 87 or fewer steps; quartile 2, 88 to 101 steps; quartile 3, 102 to 114 steps; and quartile 4, 115 steps or more. Then, because of the real-life data limitations (eg, a low number of participants for 4-group analyses, decreasing statistical power) and to potentiate the possibility of finding differences despite these limitations, data from the extreme groups (ie, inactive and very active and quartiles 1 and 4) were used for the study analyses (data and comparisons for all 4 groups can be obtained by contacting the authors).

Isolated cardiovascular risk factors were BMI, waist circumference, fasting plasma glucose, total cholesterol, and systolic and diastolic blood pressures. Global cardiovascular risk was calculated for each subject by the clustered Z score of all factors. For that, each factor value was transformed into a z value, considering the sex of the volunteer, and then all z values were summed (Z = z BMI + z waist circumference + z glucose + z total cholesterol + z systolic blood pressure + z diastolic blood pressure).

The normality of the data was verified by the Shapiro-Wilk test. The effects of the intervention in the whole sample were assessed by comparing preintervention and postintervention values with paired *t* tests. To analyze whether the effects of the intervention were influenced by the initial PA or CF levels, results of the inactive and very active PA groups were compared by mixed 2-way analysis of variance (ANOVA) and results of the quartiles 1 and 4 of CF were compared by mixed 2-way analysis of covariance (ANCOVA). In these analyses, group was used as a between main factor and moment (preintervention and postintervention) as the within main factor. In the CF analyses, age was included as a covariable because it differed between quartiles 1 and 4. Bonferroni post hoc test was used when necessary. Analyses were conducted with IBM SPSS Statistics version 19 (IBM Corp). Statistical significance was established as *P* < .05. Data are presented as mean (standard deviation).

## Results

From October 2001 through October 2015, a total of 1,592 people participated in the project. Of them, 1,466 were aged 40 to 80 years. Of those, 416 returned to the postintervention evaluation, 197 of them did the reevaluation within 3 to 6 months, and 152 reported to have followed the prescription. Thus, 152 participants formed the final sample of this study (Figure). Six participants were not assessed regarding PA level, and for 21 participants data for CF from the 2-minute step test could not be assessed because of technical problems.

**Figure:**
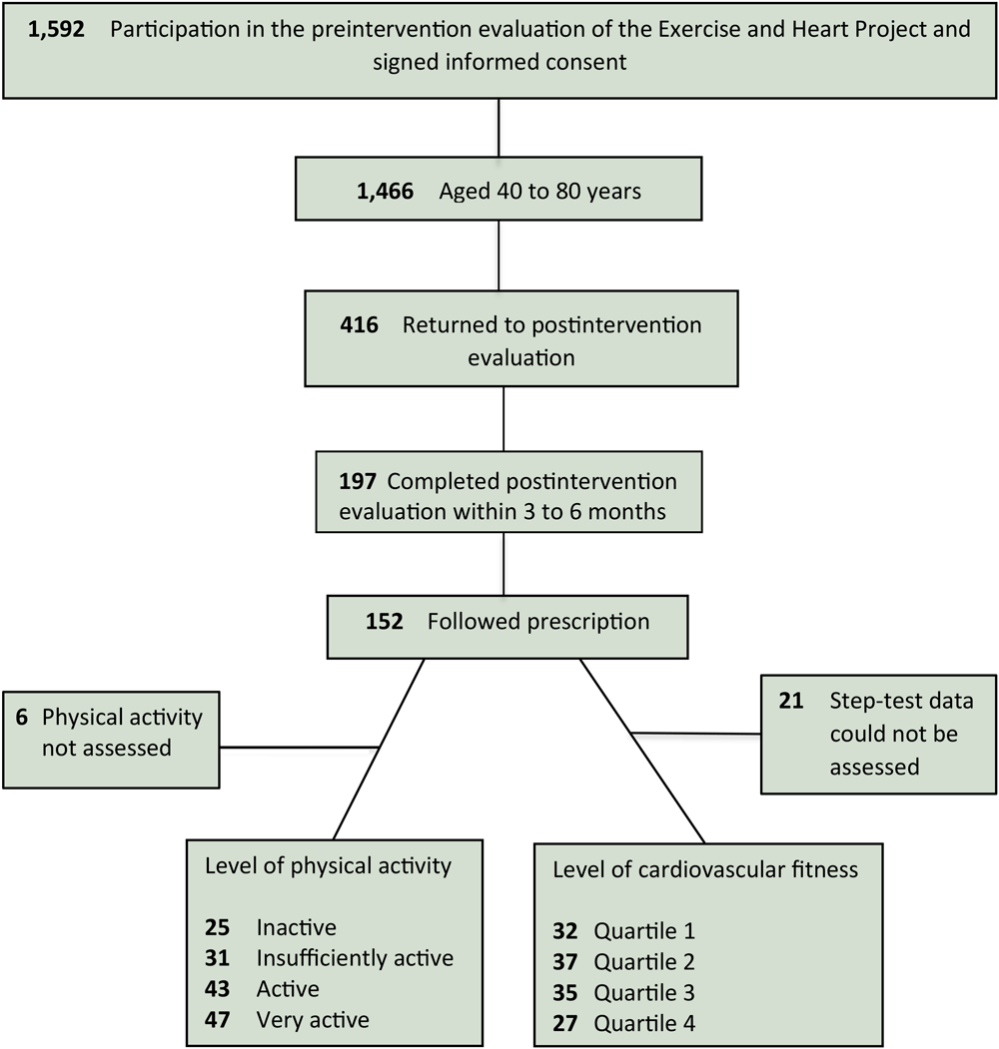
Flow of participants in the Exercise and Heart Project study of physical activity and cardiorespiratory fitness, São Paulo, Brazil, October 2001–October 2015. Levels of physical activity were defined as 1) inactive, 0 minutes per week; 2) insufficiently active, 1 to 149 minutes per week; 3) active, 150 to 299 minutes per week; and 4) very active, 300 or more minutes per week (6). Cardiorespiratory fitness level was assessed by the 2-minute step-test and classified based on quartiles of the total sample as follows: quartile 1, 87 or fewer steps; quartile 2, 88 to 101 steps; quartile 3, 102 to 114 steps; and quartile 4, 115 steps or more.

Most of the sample consisted of women and people aged 60 years or older (Table 1). Some of them reported cardiovascular diseases, and the frequency of cardiovascular risk factors was high, with weight excess (overweight and obesity) and central obesity as the most frequent factors, followed by hypertension and hypercholesterolemia. Regarding PA, 25 participants were considered inactive, 31 insufficiently active, 43 active, and 47 very active. Regarding CF, 32 participants were classified in quartile 1, 37 in quartile 2, 35 in quartile 3, and 27 in quartile 4.

**Table 1:** Sample Characteristics Assessed in the Preintervention Evaluation in the Whole Sample and in Participants Divided by the Physical Activity Level^a^ and by the Quartiles of Cardiorespiratory Fitness^b^, Data Collected in São Paulo, Brazil, October 2001–October 2015

Characteristic	Whole Sample	Physical Activity Level				Cardiorespiratory Fitness Level, by Quartile			
		Inactive	Insufficiently Active	Active	Very Active	1	2	3	4
**No. of participants**	152	25	31	43	47	32	37	35	27
**Sex, %**									
Female	74	76	80	81	57	71	83	80	63
Male	26	24	19	18	42	28	16	20	37
**Age, %, y**									
40–59	29	28	26	28	28	6	13	40^c^	45^c^^,^ d
≥60	71	72	74	72	71	94	81	60^c^	55^c^^,^ d
**Self-reported health status, %**									
Heart disease	11	8	13	14	6	13	16	6	11
Smoker	3	0	0	2	8	0	3	3	7
Weight excess	64	64	61	60	70	63	62	71	74
Central obesity	59	84	81	79	83	66	65	57	67
Hypertension	37	32	42	46	30	53	38	26	48
Hypercholesterolemia	34	28	42	37	25	44	46	29	22
Diabetes	16	16	13	19	15	19	5	20	22

a Levels of physical activity were defined as 1) inactive, 0 minutes per week; 2) insufficiently active, 1 to 149 minutes per week; 3) active, 150 to 299 minutes per week; and 4) very active, 300 or more minutes per week (6).

b Cardiorespiratory fitness level was assessed by the 2-minute step-test and classified based on quartiles of the total sample as follows: quartile 1, 87 or fewer steps; quartile 2, 88 to 101 steps; quartile 3, 102 to 114 steps; and quartile 4, 115 steps or more.

c Significantly different from quartile 1 (*P* < .05, χ^2^ test).

d Significantly different from quartile 2 (*P* < .05, χ^2^ test).

### Effects of the intervention in the whole sample

CF increased significantly from preintervention to postintervention evaluation (mean [standard deviation], 99 [19] steps vs 110 [21] steps, *P* < .001), while BMI, waist circumference, and systolic blood pressure decreased significantly (BMI: 26.3 [3.3] vs 26.1 [3.2], *P* = .02; waist circumference: 93.8 [9.9] cm vs 92.7 [8.9] cm, *P* = .01; and systolic blood pressure: 125.7 [15.5] mm Hg vs 123.2 [14.4] mm Hg, *P* = .01). Diastolic blood pressure, blood glucose, and total cholesterol did not change (diastolic blood pressure: 76.9 [8.8] mm Hg vs 75.9 [7.1] mm Hg, *P* = .07; blood glucose: 99 [11] mg/dL vs 99 [13] mg/dL, *P* = .55; and total cholesterol: 199 [31] mg/dL vs 196 [29] mg/dL, *P* = 0.46). Z score decreased significantly from preintervention to postintervention evaluation (0.15 [2.84] vs −0.52 [2.60]; *P* < .001).

### Influence of initial PA level

Comparison between inactive and very active groups showed they had similar characteristics in the preintervention evaluation (Table 1). None of the variables presented a significant interaction in the ANOVAs, but significant moment main factor effects were found for CF, BMI, waist circumference, systolic blood pressure, and Z score (Table 2). Thus, regardless of the group (inactive or very active), CF increased significantly, while BMI, waist circumference, systolic blood pressure, and Z score decreased significantly from preintervention to postintervention evaluation, while blood glucose, total cholesterol, and diastolic blood pressure did not change.

**Table 2:** Cardiorespiratory Fitness (CF) and Cardiovascular Risk Assessed Preintervention and Postintervention in Participants Classified as Inactive and Very Active^a^, Data Collected in São Paulo, Brazil, October 2001–October 2015

Category	Inactive			Very Active			ANOVA^b^ *P* Value		
	No.	Preintervention, Mean (SD)	Postintervention, Mean (SD)	No.	Preintervention, Mean (SD)	Postintervention, Mean (SD)	Group	Moment	Interaction
**CF, no. steps**	19	96.9 (20.0)	105.4 (13.0)^c^	41	103.1 (16.3)	113.0 (16.5)^c^	.27	<.001	.65
**BMI^d^**	22	25.9 (2.4)	25.7 (2.9)^c^	45	26.5 (2.7)	26.2 (2.6)^c^	.46	.02	.39
**WC, cm**	23	94.0 (10.3)	91.8 (8.9)^c^	45	96.1 (9.1)	95.0 (8.8)^c^	.72	.002	.37
**Blood glucose, mg/dL**	19	98.1 (11.4)	97.4 (10.0)	41	99.4 (11.4)	98.6 (11.1)	.60	.27	.67
**TC, mg/dL**	18	195.9 (32.2)	189.9 (25.0)	35	189.8 (26.7)	191.2 (26.8)	.84	.16	.88
**SBP, mm Hg**	23	124.4 (12.7)	119.3 (13.3)^c^	44	127.5 (13.6)	125.1 (14.9)^c^	.27	.008	.30
**DBP, mm Hg**	22	77.5 (9.6)	76.3 (6.6)	43	78.0 (8.4)	76.3 (6.8)	.66	.07	.76
**Z score**	25	0.05 (3.05)	−0.85 (2.29)^c^	47	−0.61 (2.38)	−0.26 (2.51)^c^	.66	<.001	.89

Abbreviations: BMI, body mass index; DBP, diastolic blood pressure; SBP, systolic blood pressure; TC, total cholesterol; WC, waist circumference.

a Inactive, 0 minutes per week; very active, 300 or more minutes per week (6).

b Comparisons by 2-way mixed analysis of variance (ANOVA).

c Significantly different from preintervention (*P* < .05).

d Calculated as weight in kilograms divided by the square of height in meters.

### Influence of initial CF

Comparisons between quartile 1 and quartile 4 groups showed they had similar initial characteristics except for age, which was significantly higher in quartile 1 (Table 1). For CF, the ANCOVA revealed a significant interaction (*P* < .001), showing that CF was greater in quartile 4 than quartile 1 in the preintervention evaluation and increased significantly from preintervention to postintervention in both groups with a greater increase in quartile 1 than quartile 4 (an increase of mean [SD] of 22 [14] steps vs 6 [9] steps, *P* < .001) (Table 3). For all cardiovascular risk variables, ANCOVAs did not detect any significant interaction, but significant main factor moment effects were detected for BMI, waist circumference, and Z score (Table 3), showing that regardless of the group (quartile 1 or quartile 4), these variables decreased significantly from preintervention to postintervention evaluation. Additionally, for glucose and systolic blood pressure, ANCOVA identified significant mean effects for the main factor group, showing that regardless of moment (preintervention or postintervention), quartile 4 had significantly lower glucose and systolic blood pressure than quartile 1 did.

**Table 3:** Cardiorespiratory Fitness (CF) and Cardiovascular Risk Assessed Preintervention and Postintervention in Participants Classified in the First and Fourth Quartiles of CF^a^, Data Collected in São Paulo, Brazil, October 2001–October 2015

Risk	Quartile 1			Quartile 4			ANCOVA^b^ *P* Value		
	No.	Preintervention, Mean (SD)	Postintervention, Mean (SD)	No.	Preintervention, Mean (SD)	Postintervention, Mean (SD)	Group	Moment	Interaction
**CF, no steps**	30	73.8 (8.4)	95.6 (12.5)^c^	25	120.6 (5.2)^d^	126.2 (10.0)^c^^,^ d	<.001	<.001	<.001
**Body mass index^e^**	30	26.2 (2.9)	26.0 (3.1)^c^	27	27.7 (3.0)	27.3 (3.2)^c^	.27	.008	.25
**WC, cm**	25	95.6 (8.5)	93.7 (7.6)^c^	27	96.7 (9.7)	94.8 (9.6)^c^	.95	.008	.66
**Blood glucose, mg/dL**	25	104.5 (10.6)	102.1 (10.3)	23	98.9 (10.0)^d^	97.7 (11.1)^d^	.03	.90	.95
**TC, mg/dL**	18	191.1 (25.5)	180.7 (17.7)	20	194.2 (26.0)	191.8 (30.5)	.45	.23	.37
**SBP, mm Hg**	32	129.7 (14.1)	125.1 (11.3)	24	118.4 (8.6)^d^	116.3 (11.1)^d^	.014	.09	.87
**DBP, mm Hg**	30	77.2 (8.8)	75.9 (7.4)	25	76.4 (8.1)	74.0 (7.4)	.20	.07	.60
**Z score**	32	0.97 (2.77)	−0.39 (2.34)^c^	27	0.07 (2.39)	−0.73 (2.73)^c^	.20	.001	.45

Abbreviations: CF, cardiorespiratory fitness; DBP, diastolic blood pressure; SBP, systolic blood pressure; TC, total cholesterol; WC, waist circumference.

a Cardiorespiratory fitness level was assessed by the 2-minute step-test and classified based on quartiles of the total sample as follows: quartile 1, 87 or fewer steps; quartile 4, 115 steps or more.

b Comparisons by 2-way mixed analysis of covariance (ANCOVA).

c Significantly different from preintervention (*P* < .05).

d Different from quartile 1 (*P* < .05).

e Calculated as weight in kilograms divided by the square of height in meters.

## Discussion

The main finding of our study was that the proposed interventional program increased CF and reduced BMI, waist circumference, systolic blood pressure, and global cardiovascular risk. Additionally, the increase in CF was greater in participants with lower initial CF, but the effects on cardiovascular risk factors did not differ between the participants with extreme different initial levels of PA or CF.

The high frequency of women and older adults observed in the sample of our study is a common characteristic of the adult population who visits public parks in Brazil (8). Another characteristic of the sample was the high frequency of participants with cardiovascular risk factors that are similar to frequencies reported in the Brazilian adult population (18) and suggest that projects aiming to promote PA in public parks need to include cardiovascular risk assessment before participation.

The final sample of the study (152 people) consisted of about 10% of the participants aged 40 to 80 years who joined the project (n = 1,466). It should be noted that 28% (416) of these participants returned to the postintervention evaluation but some of them did so after more than 6 months or had not followed the prescription. A low returning rate is usually reported in unsupervised studies, varying from 10% to 50% (12). The low adherence observed in this study may reflect the characteristics of a real-life program that takes place in a park (ie, no official link with the program and participants do not pay for it) or reflect the study criteria (age limitation, time between evaluations, and necessity to follow the prescription), or both.

Our results showed that the proposed interventional program was effective because it improved the main target of an aerobic intervention, CF, by about 11.1%, which is within the range expected for walking programs (ie, a mean increase of 9%) (19).

Considering its effects on cardiovascular risk, the relevance of reducing BMI and central obesity is of note now based on the epidemic obesity scores observed worldwide and in Brazil (2,18). Previous studies with supervised (11,20,21) and unsupervised (12) PA as well as with walking programs (19,22) have already shown positive effects on body composition. Thus, our results expanded previous knowledge by demonstrating that this benefit can also be achieved with a real-life interventional program, individually prescribed and executed with limited supervision, that can be easily implemented in public parks.

The absence of reduction in glucose and total cholesterol is in accordance with some previous studies that investigated both supervised and semisupervised training (20). PA seems to have little effect on glycemia in participants who did not have diabetes (23), and only a small part of the sample (16%) had diabetes. Regarding cholesterol, the effects of PA are more evident in its subfractions (especially high-density lipoprotein cholesterol) (20) that were not investigated in this study.

When analyzing blood pressure, systolic levels decreased with the intervention (approximately −3 mm Hg) while diastolic blood pressure did not change. These responses are also expected since a meta-analysis reported a mean reduction of −3.2 mm Hg (95% CI, −5.0 to −1.3 mm Hg) for systolic and no change for diastolic blood pressure after aerobic training (24).

The intervention decreased global cardiovascular risk. As the combination of risk factors, even at low levels, results in a greater cardiovascular risk than that given by the single sum of the factors (16), the reduction of global cardiovascular risk has an important health impact. Other studies have also reported reductions in global cardiovascular risk with supervised (20,21), semisupervised (25), and walking (22) programs. However, in this study, the reduction in global cardiovascular risk was greater than 400% (ie, a reduction of 0.67 Z score from a preintervention value of 0.15 to a postintervention value of −0.52), showing the strong impact that interventions like this may have on public health.

On the basis of the principle of trainability (26), one hypothesis of this study was that participants with higher initial levels of PA and CF would have lower responses to the intervention. Accordingly, CF increased less in participants initially classified as quartile 4 than as quartile 1. On the other hand, responses of CF were not different between the participants with different PA levels. It is possible that participants classified as very active, despite having a high volume of weekly PA, perform this activity with low intensity, and the individualized prescription of intensity may have promoted improvements in CF as great as that obtained in the inactive group.

Regarding cardiovascular risk, contrary to the hypothesis, the effects of the intervention were similar in the participants with extreme high and low levels of PA and CF. The absence of influence may be explained by the fact that effects of training on these factors are mainly affected by other aspects, such as genetic variability (5), age, sex, health status (20), and especially by the initial levels of these factors.

Two characteristics of the proposed intervention, individualized prescription and supervision of initial training sessions, may have resulted in the similar efficacy in participants with different levels of CF and PA. These characteristics may have made the walking training appropriate to the initial fitness level of each participant, potentiating the training effects. Additionally, the similar efficacy observed in different participants amplifies the applicability of the proposed intervention because it can benefit all users of public parks independently of their initial condition.

Because this study investigated a real-life park-based interventional program that began in 2001, a set of obstacles and difficulties was present in controlling variables, which impose limitations inherent to this study design. The sample was formed by participants who voluntarily joined the project and not a research project. Because the study intends to evaluate efficacy and not effectiveness, it included only those who came to preevaluations and postevaluations, did not change medication, and said that they followed, at least in part, the prescription. Thus, results may be different in participants who do not have these characteristics, and future studies should employ an intention-to-treat approach to investigate the intervention effectiveness. Considering the measurements, PA was assessed by a structured interview. A direct assessment with accelerometers allows greater accuracy (27); however, this kind of measure was unfeasible in a real-life park-based project that began in 2001, and other standardized questionnaires also have limitations (28). The same interview was used throughout the years of the project, which minimizes the constraint imposed. Additionally, despite the importance of different PA domains (29), only leisure time PA and weekly volume were assessed. Exercise intensity and stretching exercises execution were not controlled. Different intensities may affect the results, although stretching is not supposed to change cardiovascular health or fitness. Postintervention evaluations were conducted within 3 to 6 months, and results may be different with interventions lasting for different periods. Future studies should consider evaluating PA intensity, other kinds of PA, and shorter- and longer-lasting interventions. It was not possible to have a control group without exercise, because data derived from a real-life project. Thus, several intervening variables were neither controlled nor evaluated. However, as real-life PA interventions are rarely formally evaluated, the practical application of our results is remarkable. Finally, comparisons were performed only between the extreme groups of PA and CF. The absence of differences between these groups make it very improbable that differences can be detected with the inclusion of the middle groups. In accordance, a complementary comparison among the 4 groups (data not shown can be obtained by contacting the authors) also revealed an absence of influence of the initial CF and PA levels on responses to the intervention. Future studies with bigger samples should include more group comparisons.

A real-life park-based interventional program including individualized prescription of walking and limited supervision of execution improves CF and decreases cardiovascular risk in adult users of a public park. Additionally, its effects in decreasing cardiovascular risk is independent of the participant’s initial level of PA and CF.

## Post-Test Information

To obtain credit, you should first read the journal article. After reading the article, you should be able to answer the following, related, multiple-choice questions. To complete the questions (with a minimum 75% passing score) and earn continuing medical education (CME) credit, please go to http://www.medscape.org/journal/pcd. Credit cannot be obtained for tests completed on paper, although you may use the worksheet below to keep a record of your answers.

You must be a registered user on http://www.medscape.org. If you are not registered on http://www.medscape.org, please click on the “Register” link on the right hand side of the website.

Only one answer is correct for each question. Once you successfully answer all post-test questions, you will be able to view and/or print your certificate. For questions regarding this activity, contact the accredited provider, CME@medscape.net. For technical assistance, contact CME@medscape.net. American Medical Association’s Physician’s Recognition Award (AMA PRA) credits are accepted in the US as evidence of participation in CME activities. For further information on this award, please go to https://www.ama-assn.org. The AMA has determined that physicians not licensed in the US who participate in this CME activity are eligible for AMA PRA Category 1 Credits™. Through agreements that the AMA has made with agencies in some countries, AMA PRA credit may be acceptable as evidence of participation in CME activities. If you are not licensed in the US, please complete the questions online, print the AMA PRA CME credit certificate, and present it to your national medical association for review.

## Post-Test Questions

### Study Title: Effects of a Real-Life Park-Based Physical Activity Interventional Program on Cardiovascular Risk and Physical Fitness

#### CME Questions

1. Which of the following variables followed in the current study improved from pre-intervention to postintervention in the cohort who exercised?
  - Systolic blood pressure (SBP)
  - Diastolic blood pressure
  - Blood glucose
  - Total cholesterol
2. How did baseline levels of physical activity affect the outcomes of the current study?
  - Only highly active participants experienced an improvement in cardiovascular biomarkers
  - Only inactive participants experienced an improvement in cardiovascular biomarkers
  - Cardiometabolic biomarkers improved regardless of baseline physical activity
  - Cardiometabolic biomarkers did not significantly change with the exercise intervention regardless of baseline activity level
3. How did cardiorespiratory fitness levels change from the preintervention to the postintervention periods in the current study?
  - Cardiorespiratory fitness increased only among participants with high baseline fitness levels
  - Cardiorespiratory fitness increased only among participants with low baseline fitness levels
  - Cardiorespiratory fitness increased to a greater degree during the exercise intervention among participants with high baseline fitness levels
  - Cardiorespiratory fitness increased to a greater degree during the exercise intervention among participants with low baseline fitness levels
4. Which of the following statements regarding cardiorespiratory fitness at baseline and intervention effects on cardiometabolic biomarkers is most accurate?
  - Only adults with high baseline fitness experienced a reduction in SBP at the end of the intervention
  - Only adults with high baseline fitness experienced a reduction in waist circumference at the end of the intervention
  - Only adults with low baseline fitness experienced a reduction in SBP at the end of the intervention
  - Baseline cardiorespiratory fitness did not significantly affect the effects of the exercise intervention on cardiometabolic biomarkers
